# Climate-responsive vector control strategies for *Aedes albopictus*

**DOI:** 10.1186/s13071-025-06791-2

**Published:** 2025-05-11

**Authors:** Jesús Bellver-Arnau, Laura Blanco-Sierra, Santi Escartin, Simone Mariani, Frederic Bartumeus

**Affiliations:** 1 Departament d’Ecologia i Complexitat, Centre d’Estudis Avançats de Blanes (CEAB), Blanes, Spain; 2 Centre de Recerca Ecològica i Aplicacions Forestals (CREAF), Cerdanyola del Vallès, Spain; 3 Institució Catalana de Recerca i Estudis Avançats (ICREA), Barcelona, Spain

**Keywords:** Climate sensitive pest management, Mosquito control, Invasive species, *Aedes albopictus*, Public health

## Abstract

**Background:**

The rise in mosquito-borne diseases such as dengue, Zika, and chikungunya, exacerbated by the ever-expanding habitats of *Aedes albopictus*, poses a significant public health risk. Even marginal improvements in vector control efficacy can be crucial in mitigating these risks.

**Methods:**

In this study, we employed a metapopulation model to simulate *Ae. albopictus* population dynamics and dispersal, optimizing the timing and spatial allocation of larvicidal treatments.

**Results:**

Simulations revealed that larvicide treatments are most effective when applied preventively, early in the mosquito season, particularly under conditions of lower-than-average cumulative rainfall and, to a minor extent, colder-than-average temperatures, as these conditions limit larvae proliferation. We found that breeding site characteristics, particularly surface area and maximum water holding capacity, are critical in determining optimal treatment allocation in scarce-resource scenarios. However, a cost-effectiveness trade-off exists, as larger breeding sites offer more substantial reductions in mosquito populations but also demand higher larvicide dosages. Spatial factors such as breeding site distribution had minimal impact on treatment efficacy, possibly due to the high mobility range of adult mosquitoes compared with the size of the study area.

**Conclusions:**

Our results highlight the superior efficiency of the optimized approach in comparison with routine vector control strategies, especially when resources are limited, offering a more effective use of larvicide in controlling mosquito populations. This study demonstrates that vector control strategies for *Ae. albopictus* can be significantly enhanced by considering climatic variables and breeding site characteristics in treatment planning. This research provides a framework for developing cost-effective and flexible mosquito control programs that can adapt to environmental conditions, potentially improving public health outcomes by reducing the transmission risk of mosquito-borne diseases.

**Graphical Abstract:**

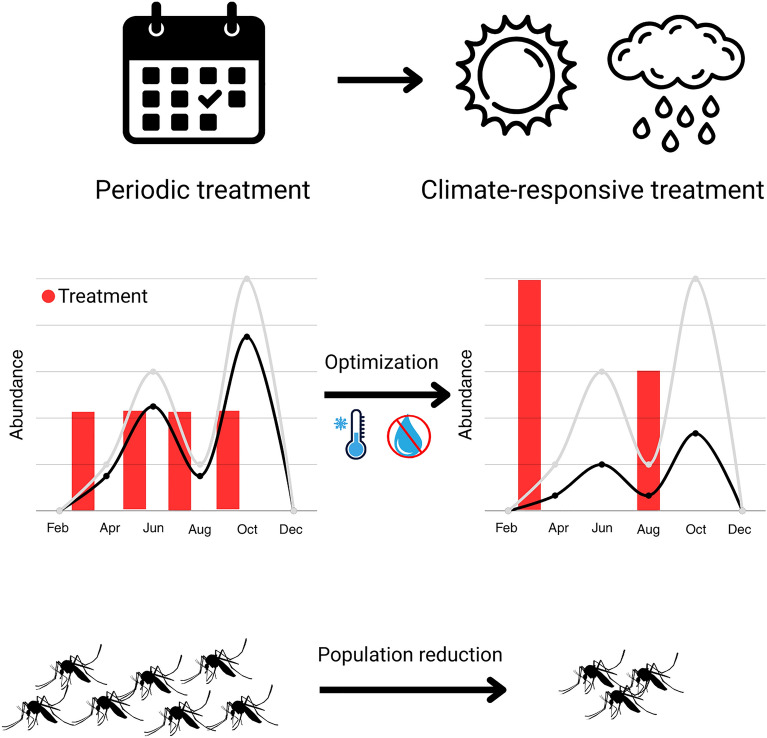

**Supplementary Information:**

The online version contains supplementary material available at 10.1186/s13071-025-06791-2.

## Background

The global increase in mosquito-borne diseases such as dengue and chikungunya [1, 2], coupled with the rapid habitat expansion of their primary vectors, *Aedes albopictus* and *Aedes aegypti* [3], has become a significant concern in recent years. Notably, *Ae. albopictus* has demonstrated exceptional genetic, ecological, behavioral, and physiological adaptability [4], enabling it to colonize new, more temperate regions.

In Europe, *Ae. albopictus* has been expanding at an alarming rate over the past two decades and has firmly established itself along the Mediterranean coast [5, 6]. The presence of *Ae. albopictus* in these regions carries significant epidemiological implications, leading to the autochthonous transmission of dengue and chikungunya [7, 8], and resulting in outbreaks in countries such as Croatia [9], France [10], and Italy [11]. In particular, in the Spanish region of Catalonia, where the study area of this work is located, 11 autochthonous dengue cases were reported in the last 2 years [12].

Given that vector control remains the most effective strategy for combating vector-borne diseases [13], the development of more efficient control methods is crucial in the current context of increasing disease risk. Vector control can target adult mosquitoes directly (e.g., through insecticide spraying or rear-and-release techniques) or focus on the immature stages at mosquito breeding sites [13]. In this study, we concentrate on the latter approach, specifically, we focus on biological control methods using the larvicidal product Vectomax^®^ FG, which combines *Bacillus thuringiensis* var. *israelensis* and *Lysinibacillus sphaericus*. The toxins produced by these bacteria are highly effective against *Ae. albopictus* larvae [14], but mostly harmless for other fauna inhabiting the water drains [15].

Studies indicate that seasonality plays a crucial role in shaping the abundance and biodiversity of mosquito populations in both urban and semi-urban environments, such as parks and public gardens [16, 17]. Consequently, variations in the timing of treatment applications inevitably lead to differences in their overall efficacy. Moreover, mosquitoes tend to prefer certain breeding sites based on various factors such as location, site characteristics, and the surrounding environment [18]. This suggests that a more targeted allocation of resources, such as prioritizing treatments at breeding hotspots [19], could enhance control efficiency. Therefore, any integrated vector management plan must consider the spatio-temporal dynamics of mosquito populations to maximize effectiveness and cost-efficiency [20, 21].

This study aims to optimize larvicide treatment strategies for the Marimurtra Botanical Garden in Blanes, Spain, by determining the most effective timing and spatial targeting of interventions. Using a metapopulation model, we simulated mosquito population dynamics and dispersal across the garden throughout the season. Through numerical simulations, we refined treatment strategies to minimize adult mosquito presence year-round. Our analysis identified temporal and spatial patterns that could improve current control measures. We explored correlations between optimal treatment timings and weather variables, such as temperature and rainfall, that could serve as early warnings. In addition, we identified intrinsic characteristics of breeding sites, such as maximum volume and relative position, to prioritize treatment efforts and reduce the need for costly trial and error.

## Methods

### Study site

The study was conducted at the Jardí Botànic Marimurtra in Blanes, Spain, a four-hectare botanical garden divided into three interconnected sections. The garden is highly susceptible to mosquito infestations, particularly by *Ae. albopictus*, due to its extensive network of water management systems, such as storm and sewage drains, which create ideal breeding sites. In addition, the presence of bromeliads, plantains, and ornamental ponds further supports mosquito larvae. With over 4000 plant species, many of which provide food and shelter for adult mosquitoes, and a constant presence of workers and visitors offering a reliable blood source, the garden presents a highly conducive environment for mosquito proliferation. The presence of visitors from different parts of the world (more than 100,000 per year) makes mosquito control a major concern for the botanical garden, not only to prevent nuisances but also reduce disease transmission risks.

Current mosquito control measures at the Jardí Botànic Marimurtra focus primarily on applying larvicide to the breeding sites. These measures are outsourced to a private company, independently of the current study. This work attempts to follow and improve the preexisting treatment practices. The larvicide used is a blend of the biological agents *Bacillus thuringiensis* var. *israelensis* and *Lysinibacillus sphaericus*, commercialized under the name Vectomax^®^ FG. Secondary control actions include the mechanical removal of natural or artificial breeding sites through cleaning or refurbishing problematic water drains and introducing *Gambusia affinis*, a natural predator of mosquito larvae, into ornamental ponds.

**Fig. 1:**
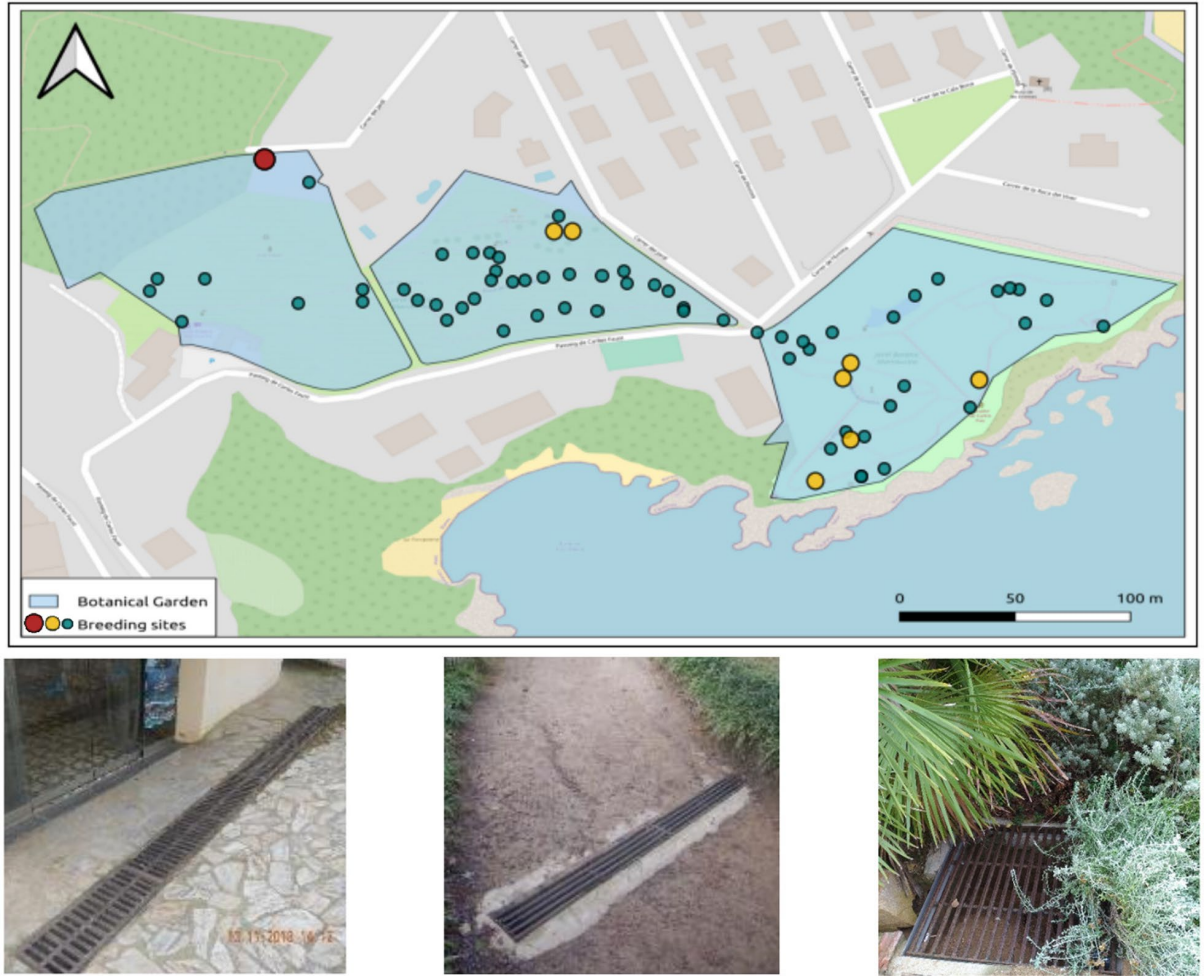
Map of the study area and the location of the selected breeding sites within it (top). Photos of actual breeding sites considered in each garden (bottom). The color of the dots indicates the dose at which breeding sites are treated (30 g for red, 20 g for yellow, and 10 g for green)

We identified 195 potential breeding sites within the botanical garden and selected 70 for this study based on historical larval activity. To correlate optimal larviciding with weather conditions, we focused on sites with water dynamics influenced by rainfall, excluding fountains and permanent small ponds. We also excluded sites treated with methods other than Vectomax^®^ FG, such as bromeliads and plantain species sprayed with Gnatrol^®^ SC (*Bacillus thuringiensis* var. *israelensis*). Most selected sites were approximately rectangular water drains (storm and sewage), though we also included a few other artificial structures that retain water after rainfall. The distribution of these sites is shown in Fig. 1.

### Mathematical modeling of breeding site dynamics

Let us consider the following metapopulation system modeling mosquito population dynamics under larvicidal control,

1 $$\begin{aligned} \begin{aligned} \frac{dL_i}{dt}&= \alpha (T) A_i - (\mu (T) + \delta ^L(T))L_i -\frac{L_i^2}{K_i(R)} - \tau \frac{c_iL_i}{V_i(R)}, \\ \frac{dA_i}{dt}&= \frac{1}{2}\mu (T) L_i- \delta ^A(T) A_i - \sum _{j=1}^{N} m_{ij}(A_i-A_j) \\ \frac{dc_i}{dt}&= - \kappa _c c_i - \kappa _L \frac{c_iL_i}{V_i(R)}, \quad i=1,\dots ,N. \end{aligned} \end{aligned}$$

This system, models an area with *N* distinct breeding sites., each characterized by three key variables:

- $L_i$, the larval population at breeding site *i*, influenced by environmental factors (water availability and temperature) and larvicide application.
- $A_i$, the adult mosquito population in the vicinity of breeding site *i*, determined by larval development into adults in the site and dispersal from other sites. It is affected by temperature and, indirectly, by larviciding. Predation is not explicitly considered.
- $c_i$, the amount of larvicidal product at site *i*, which impacts larval survival and, consequently, both larval and adult populations.

We analyze the interactions and dynamics of these variables in detail below.

#### Biology of the mosquito

In (1), we consider the birth rate of larvae, $\alpha$, the death rates of both larvae and adults, $\delta ^L$, $\delta ^A$, and the development rate from larva to adult, $\mu$, depending on temperature, *T*. The temperature-dependent functions used to parameterize the life cycle of *Ae. albopictus* throughout the season were obtained from [22]. As it remains unclear whether the minimum, average, or maximum daily temperature should be used when computing these parameters, we used a combination of these. We used the maximum daily temperature to compute the relevant temperature-dependent parameters that shape development and reproduction ($\mu$ and $\alpha$), while the minimum daily temperature was used for death-related parameters ($\delta ^L$ and $\delta ^A$). This choice was motivated by the range of values achieved by these parameters for the usual temperatures in the study site. For the achieved temperatures, $\mu$ and $\alpha$ present a wider variation at higher temperatures, while $\delta ^L$ and $\delta ^A$ do so at lower temperatures. Therefore, we have used the most sensitive temperature ranges expected to modulate the fluctuations of mosquito populations at the high and the low ends. We obtained the necessary data on minimum and maximum daily temperatures ($T^{min}$ and $T^{max}$ from now on) from the nearest meteorological station in Malgrat de Mar.

#### Carrying capacity of the environment and water dynamics

Larval competition is an important force shaping mosquito population dynamics inside the breeding sites [23]. This fact is reflected in our model by a carrying capacity for each site, $K_i$. Competition is driven by resource availability, which we consider directly related to water volume in the site, $V_i$. This volume, in its turn, is determined by the shape and size of the site and the amount of rainfall, *R*. We obtained data of both the surface area and the maximum water-holding capacity of the breeding sites considered, most of which were rectangular cuboids (Fig. 1), by direct measurement. The relationship between rainfall and the carrying capacity of breeding sites for different mosquito species has been modeled in various ways across the literature. These methods range from using ordinary differential equations (ODE models) [24], to considering weighted averages of rainfall over previous days [25, 26], incorporating human density factors [27], or employing more heuristic approaches [28]. Given the absence of definitive evidence favoring any specific modeling approach, we favored a straightforward approach over the more complex ones. We assumed the carrying capacity of mosquito breeding sites to be directly proportional to the volume of water they contain. In turn, we considered the water volume held by breeding sites to be proportional to their surface area and the 21-day cumulative rainfall ($R_{21}$ from now on), as it has been found to be a significant driver of mosquito abundance in previous studies in the botanical garden [16]. Once a site reaches its maximum water-holding capacity, its volume cannot increase anymore. It is important to note that results can be dependent on the shape and value of the carrying capacity, or more generally, in the way intraspecific competition is modeled, sometimes to a great extent [29].

In mathematical terms, we considered

$$\begin{aligned} V_i(R_{21})= \min \left( \nu _{R} R_{21},V^{max}_{i}\right) , \ K_{i}(R_{21})=\nu _{K}V_i(R_{21}) \end{aligned}$$

We obtained the rainfall data from the closest meteorological station (4.98 km away).

#### Dispersal of mosquitoes

We consider adult mosquitoes capable of flying unimpeded across the whole botanical garden. The dispersal of female mosquitoes from their natal breeding sites to new sites for egg-laying is modeled to occur at rates that depend on the distance between the two sites. Namely, to model the dispersal rate between two breeding sites *i* and *j*, we used the following function

$$\begin{aligned} m_{ij}=\sigma \frac{s}{\lambda }\left( \frac{d(i,j)}{\lambda }\right) ^{s-1}\exp \left[ {-\left( \frac{d(i,j)}{\lambda }\right) ^{s}}\right] , \end{aligned}$$

with *d*(*i*, *j*) being the distance between the two breeding sites. This function is based on the Weibull distribution, whose shape and scale parameters, *s* and $\lambda$, respectively, are taken from previous work based on experimental data in urban mosquito dispersion [30]. The parameter $\sigma$, capturing the strength of this dispersal, was fit from the experimental data [31, 32]. The values of these parameters are shown in Table 1.

#### Larvicide dynamics

In the model, larvicide is assumed to decay naturally at a rate $\kappa _c$, while it also diminishes due to active ingestion by larvae, which occurs at a rate $\kappa _L$. The toxicity of the product in (1) is denoted by $\tau$. We fit these parameters using data obtained in a bioassay conducted in the laboratory on Vectomax^®^ FG effect duration (Additional File 2). Given that the time required to perform a treatment is negligible compared with the overall duration of the study, we model the treatment as an impulsive control, represented mathematically as a sum of Dirac delta functions. This allows us to capture the instantaneous effect of the larvicide applications. In mathematical terms, the control at a breeding site *i* is given by $\gamma ^i \sum _k \xi _k^i\delta (t-t_k)$. Here $t_k$, $k=1,\dots ,n$ stands for the times at which treatments are applied. $\xi _k^i$ can take the values 1 and 0, $\xi ^i_k=1$ implies that larvicide has been applied at breeding site *i* at time $t_k$, while $\xi ^i_k=0$ means it has not. Finally, $\gamma ^i$ is the dosage applied at a given breeding site at each deployment. The dosage of Vectomax^®^ FG recommended by the manufacturer is 10 g/50 L, but the company in charge of the treatments usually increases this amount to ensure the efficacy of the product, even in cases of high population density or high levels of organic matter in the water [33]. In these cases the company applies a maximum of 33 g/50 L. For practical reasons, larvicide dosage is typically standardized on the basis of site size, with a fixed amount of product being applied regardless of the actual water volume present. This approach is often favored because estimating the exact water volume at each site can be challenging and time-consuming. We clustered breeding sites into three groups using a *k*-means algorithm, based on their surface area and maximum water-holding capacity. Each group was assigned a fixed dosage for treatment in the simulations: 10 g, 20 g, and 30 g, respectively. Figure 1 shows the distribution of sites across the garden.

### Control optimization

When taking into account everything exposed so far, system 1 can be rewritten as

2 $$\begin{aligned} \begin{aligned} \frac{dL_i}{dt}&= \alpha (T^{max}) A_i - (\mu (T^{max}) + \delta ^L(T^{min}))L_i -\frac{L_i^2}{K_i(R_{21})} - \tau \frac{c_i L_i}{V_i(R_{21})}, \\ \frac{dA_i}{dt}&= \frac{1}{2}\mu (T^{max}) L_i- \delta ^A(T^{min}) A_i - \sum _{j=1}^{N} m_{ij}(A_i-A_j)\\ \frac{dc_i}{dt}&= - \kappa _c c_i - \kappa _L \frac{c_iL_i}{V_i(R_{21})}, \quad t\in [0,t_f]\setminus \{t_k\}_{k=1}^{n}, \quad i=1,\dots ,N. \\ c_i(t_k^+)&= c_i(t_k^-) + \xi _k^i \gamma ^i , \quad k=1,\dots ,n. \end{aligned} \end{aligned}$$.

The impulsive control is translated into discontinuities in the amount of larvicide present in the breeding sites at times $t_k$. Some constraints on the control function have to be considered. First, we impose a minimal time between any two applications, $t_{min}$ (in our case, $t_{min}=21$ days). Also, we impose a limit on the amount of larvicide that can be applied in a treatment season, denoted by *C*. In other words, we assume

$$\begin{aligned} \sum _{i=1}^{N}\sum _{k=1}^{n}\gamma ^i \xi _k^i\le C. \end{aligned}$$

The values of the parameters in system (1) and (2) considered for the study can be found in Table 1.

Therefore, the control problem of minimizing the total amount of adult mosquitoes during a certain period $[0,t_f]$ can be stated in the following way

3 $$\begin{aligned} \inf _{\begin{array}{c} \{t_k\}_k\in {\mathcal {T}} \\ \{\xi _k^i\}_{i,k}\in \Gamma \end{array}}\int _0^{t_f}\left( \sum _{i=1}^{N}A_i(t) \right) dt, \end{aligned}$$

where

$$\begin{aligned} {\mathcal {T}}:=\left\{ \left( t_1,\dots ,t_n \right) \in [0,t_f]^n \quad | \quad t_k-t_{k-1} \ge t_{min}, \quad k=2,\dots ,n \right\} \end{aligned}$$

and

$$\begin{aligned} \Gamma :=\left\{ \xi _k^i\in \{0,1\} \quad \bigg | \quad \sum _{i=1}^{N}\sum _{k=1}^{n}\gamma ^i \xi _k^i\le C \right\} . \end{aligned}$$

### Statistical analyses

We performed several statistical tests and analysis to interpret the outcomes of our simulations and to establish the significance of the results.

We performed Wilcoxon signed-rank tests [34] to evaluate the differences between the distributions of climatic variables (i.e., temperature and cumulative rainfall) during the treatment season and at optimal treatment times.

To explore the spatial heterogeneity in the optimized treatments, we performed a logistic regression analysis [35]. We used the probability of larvicide treatment at each breeding site as the response variable and examined time-independent covariates (i.e., those that remained constant over time and across simulations). These covariates included intrinsic properties of the breeding sites, such as maximum volume, surface area, and the dosage at which they are treated. We applied the natural logarithm to these variables, as this transformation exhibited a relationship with the log-odds of the response variable closer to linearity, aligning with one of the basic assumptions of logistic regression models [35].

In addition, we incorporated measures of spatial distribution, such as: (i) the mean distance to neighboring breeding sites; (ii) the number of breeding sites within a distance smaller than $\lambda$ (the average distance traveled by adult mosquitoes used in the simulations [30]); and (iii) a measure of spatial clustering of the breeding sites. Specifically, the local clustering coefficient [36]. This was computed using Python’s *Networkx* package [37], where breeding sites were treated as vertices in a fully connected weighted graph, with edge weights corresponding to the inverse of the distance between sites. All variables were scaled before running the analysis for proper comparison.

We incorporated the amount of larvicidal product available ($C_{0.25}$, $C_{0.50}$ and $C_{0.75}$) as a random effect (random intercept) in our analysis. Simulations for $C_{max}$ were excluded from the analysis since the probability of treatment is uniformly 1 across all breeding sites in that scenario.

To identify the best-fitting minimal model, we employed a step-wise backward deletion process. Model selection was guided by the Akaike information criterion corrected for small sample sizes (AICc). During this process, combinations of covariates with high collinearity were removed to refine the model. The analysis was performed in RStudio using the *lme4* [38], *car* [39], *MuMIn* [40], and *DHARMa* [41] packages.

We performed a Moran’s *I* test [42] on the response variable (probability of larvicide treatment at each breeding site) to asses a potential spatial autocorrelation between treatments.

**Table 1:** Values of the parameters used in simulations

Climate	Parameter	Value	Source
$T^{max}$	Maximum temperature	–	Closest meteorological station
$T^{min}$	Minimum temperature	–	Closest meteorological station
$R_{21}$	21-days cumulative rainfall	–	Closest meteorological station
**Larvicide**			
$\kappa _c$	Intrinsic decay rate	0.089 $\hbox {day}^{-1}$	Fit from data
$\kappa _L$	Active removal rate	0.021 L $\cdot$ $\hbox {larvae}^{-1}$ $\cdot$ $\hbox {day}^{-1}$	Fit from data
$\tau$	Toxicity	407.66 L $\cdot$ g$^{-1}$ $\cdot$ day$^{-1}$	Fit from data
$\gamma ^i$	Product dosage	{10 g, 20 g, 30 g}	-
**Mosquito**			
$\alpha$	Birth rate	Function of $T^{max}$	[22]
$\mu$	Development rate	Function of $T^{max}$	[22]
$\delta ^L$	Larval death rate	Function of $T^{min}$	[22]
$\delta ^A$	Adult death rate	Function of $T^{min}$	[22]
$K_i$	Carrying capacity	Function of $R_{21}$	Estimated from data
$\nu _R$	Breeding sites water retention	0.75	–
$\nu _K$	Water to larvae prop. constant	106.7 larvae $\cdot$ L$^{-1}$	–
$\sigma$	Migration rate strength	12.59 m $\cdot$ day$^{-1}$	Fit from [31]
			and [32]
*s*	Mobility shape parameter	1.323	[30]
$\lambda$	Mobility scale parameter	110.3 m	[30]
**Control**			
$C_{max}$	Max. larvicide available	3240 g	–
$t_{min}$	Minimal time between treatments	21 days	–

**Table 2:** *p*-Values for the Wilcoxon signed-rank test comparing the distributions of the deviations in temperature and 21-day cumulative rainfall at the time of treatment compared with the overall background distribution

Wilcoxon signed-rank test	$C_{0.25}$	$C_{0.50}$	$C_{0.75}$	$C_{max}$
Temperature *p*-value	1.31 × 10^−56^	0.087	3.83 × 10^−5^	1.28 × 10^−65^
21 days cumulative rainfall *p*-value	9.94 × 10^−81^	2.97 × 10^−48^	3.99 × 10^−40^	6.51 × 10^−34^

**Table 3:** Logistic regression results of the best-fitting minimal model

Accuracy	68.52%		
Random effects	Variance	Std. dev.	
*C*	0.8829	0.9397	
Fixed effects	Estimate	Std. error	*p*-Value
Log maximum volume	0.11060	0.01349	2.47 ×10^−16^
Log area	0.43470	0.03273	< 2× 10^−16^
Log dose	−1.37274	0.07603	< 2 × 10^−16^
Number of observations	25200

**Table 4:** Mosquito abundance percentage reduction obtained by deploying the optimal strategies designed in this work compared with routine vector control interventions according to the years 2020, 2021, and 2022

	$C_{0.25}$	$C_{0.50}$	$C_{0.75}$	$C_{max}$
$\Delta J^{\text {rout,a}}$	−24.90%	−54.37%	−82.95%	−99.38%
$\Delta J^{\text {rout,b}}$	−31.30%	−65.81%	−92.06%	−100%
$\Delta J^{\text {opt}}$	−70.19%	−92.84%	−99.69%	−100%

**Table 5:** ${R}^2$ for exponential and linear fits for *Optimized* and *Routine* strategies and differences in the Akaike information criterion between the best fit (highlighted) and its alternative

	$R^2_{\text {exp}}$	$R^2_{\text {linear}}$	$\Delta$AIC
*Routine* (avg.)	0.935	**0.992**	28.02
*Routine* (best)	0.953	**0.963**	1.20
*Optimized*	**0.999**	0.734	10.63

## Results

### Numerical simulations

To determine optimal vector control strategies, we performed a stochastic optimization of both the timing of treatments and the selection of breeding sites for treatment. The treatment window spanned from 1 April to 31 October, aligning with the typical intervention period at the botanical garden. Within this window, we considered four treatment actions, which is consistent with the usual number of interventions conducted. We evaluated four total amounts of larvicide: $C_{max}$, which is the amount required to treat all breeding sites four times, and three other amounts $C_{p}=p\cdot C_{max}$, with $p=0.25,0.50,0.75$.

**Fig. 2:**
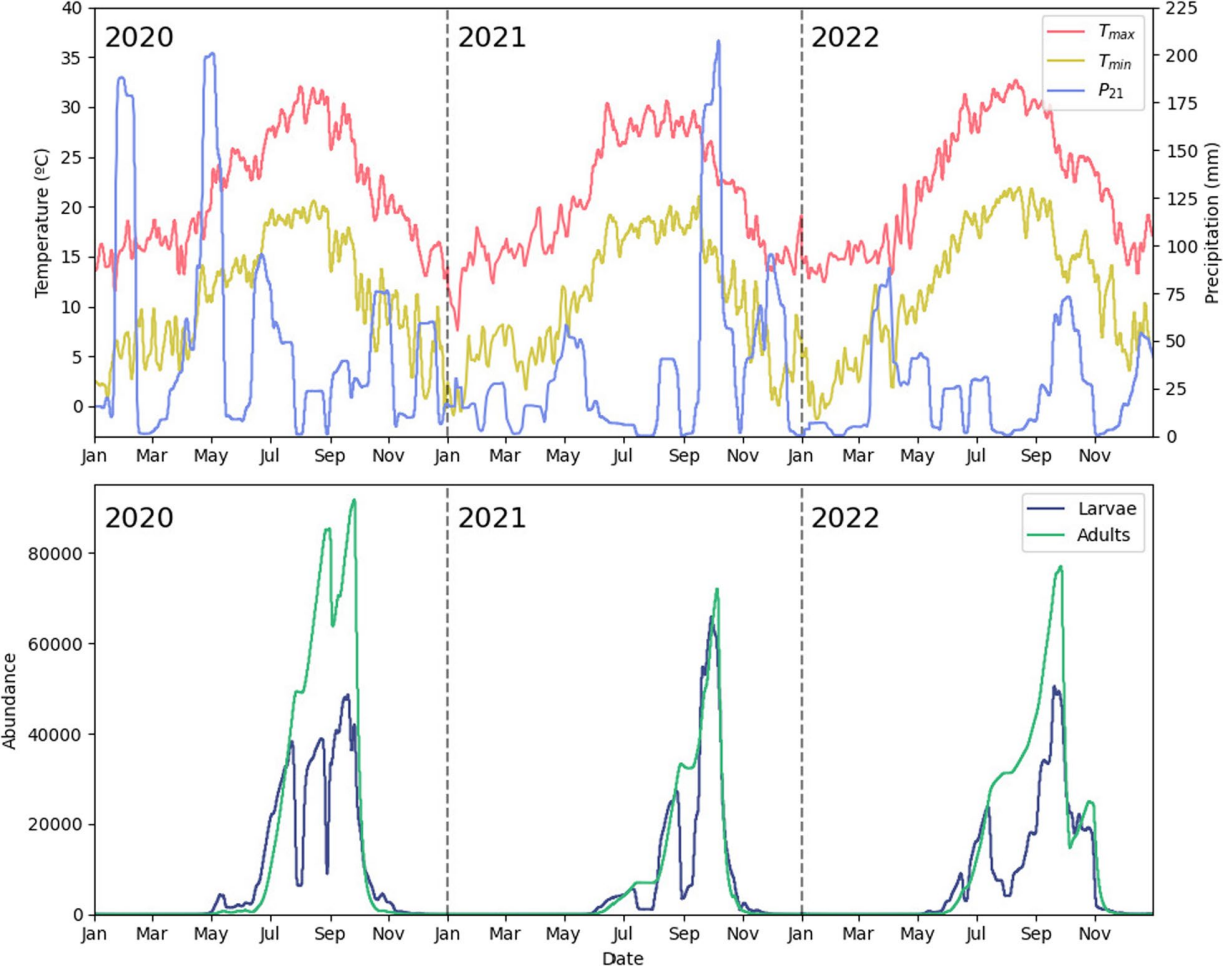
Top: relevant climatic conditions for the model during 2020–2022. Bottom: simulated larvae and adult dynamics in the garden during 2020–2022 without interventions. Each year’s simulation is independent from previous years

We employed a straightforward stochastic optimization algorithm, where, in each iteration, treatment times were randomly adjusted, and a random subset of the breeding sites treated in the previous iteration was replaced with a different subset of untreated sites. After testing up to 900 new configurations, the most effective one was selected as the basis for the next iteration. This process was repeated for 70 iterations or until a better configuration was found. Time adjustments and subset sizes were gradually reduced to fine-tune optimization. Twenty-five simulations were conducted for each year and larvicide amount *C*, selecting only the ten best-performing simulations for further analysis. Both the initial conditions for $L_i$ and $A_i$ were randomized within some reasonable bounds in each simulation (all $c_i$ were set to 0), as well as the initial times and breeding sites selected for treatment. The dynamics of system (2) without any control intervention for the years 2020–2022 can be seen in Fig. 2.

### Temporal optimization

**Fig. 3:**
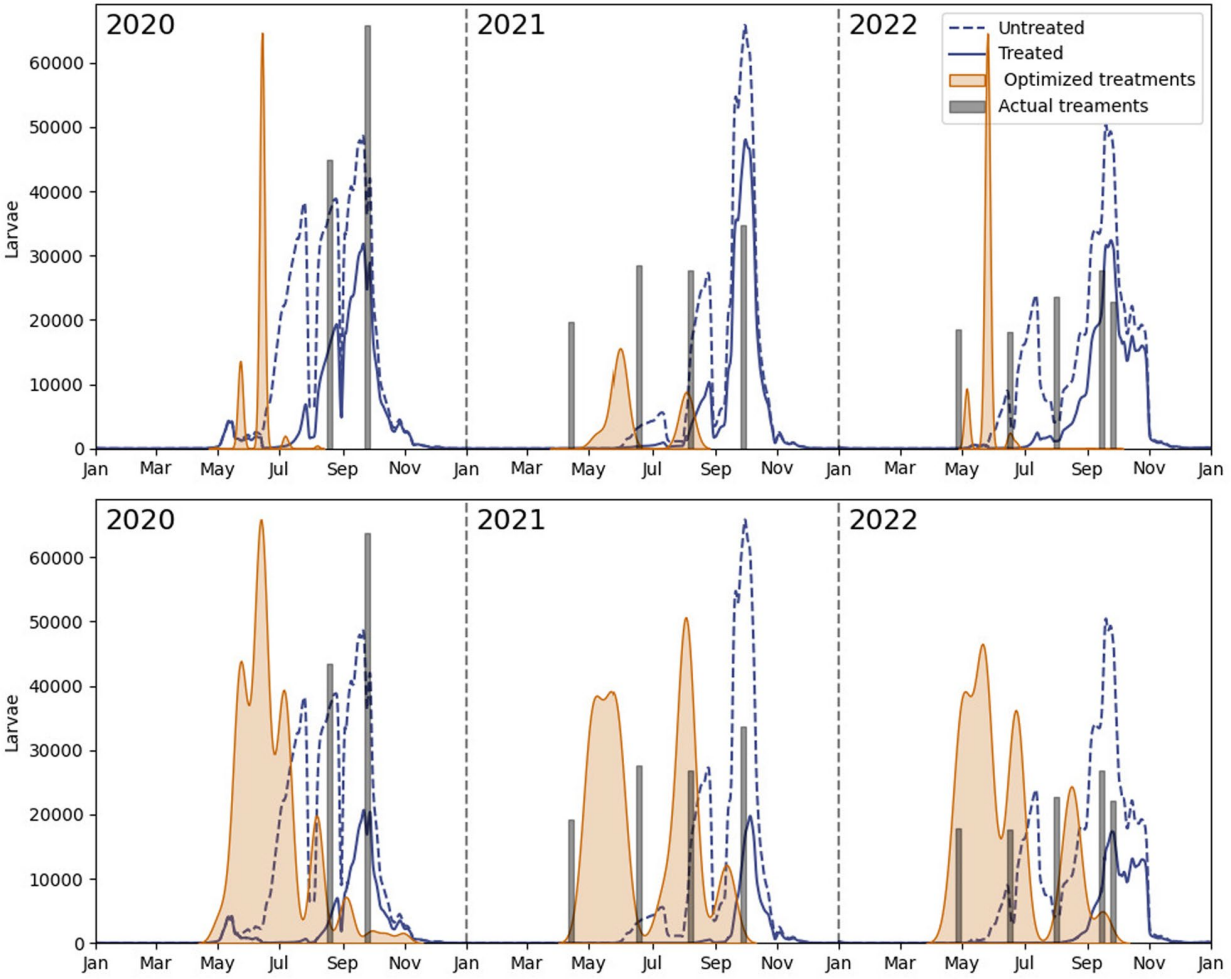
Treatment densities and average larva dynamics for $C_{0.25}$ (top) and $C_{0.50}$ (bottom). The actual distribution of treatments performed in the botanical garden was included for reference

Figure 3 shows the treatment densities (i.e., treatment allocation over time) obtained for $C_{0.25}$ and $C_{0.50}$. We applied a kernel distribution estimation (kdeplot from the Seaborn library in Python) to the larvicide amounts deployed across the ten best-performing simulations, i.e., the ten simulations with the smallest adult mosquito populations throughout the year [see Eq. (3)]. Thus, the temporal pattern observed is an average over the ten best simulations. Densities were normalized for each value of *C* separately. Our results revealed that treatments were predominantly concentrated in late spring and early summer (around June) in 2020 and 2022, while in 2021 they were more evenly split between early and late summer. For the less restrictive dosage of $C_{0.50}$ treatments were distributed more evenly throughout the season, with secondary peaks emerging in all 3 years. Notably, in every case, primary and most secondary peaks roughly coincided with either minimums or periods of rapidly increasing larval numbers in the untreated population. Detailed results for $C_{0.75}$ and $C_{max}$ are provided in Additional File 1.

**Fig. 4:**
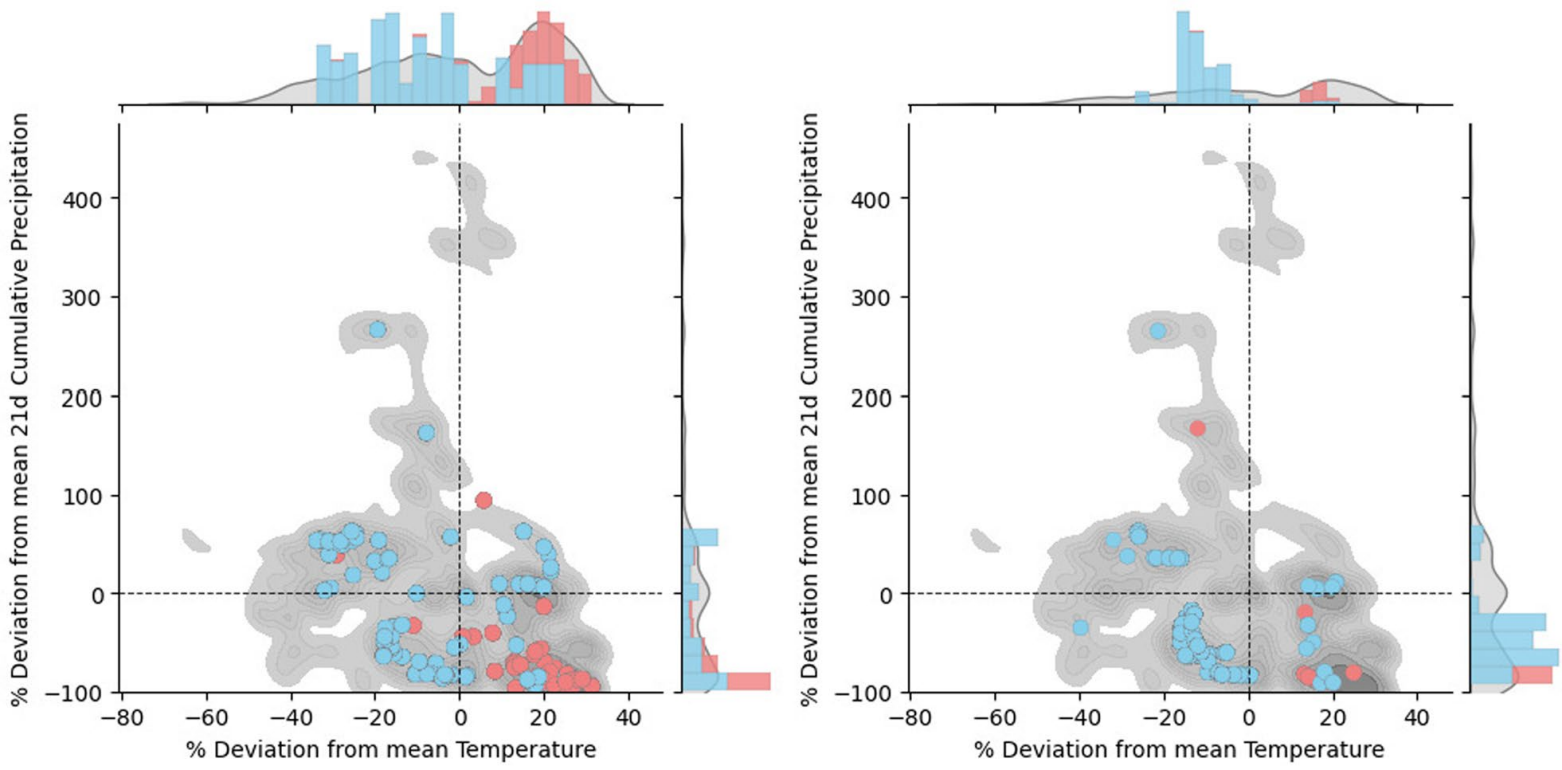
Percentage of deviation from mean temperature and mean 21-day cumulative rainfall at treatment for $C_{0.50}$ (left) and $C_{0.25}$ (right). Blue and red dots correspond with treatment times resulting from simulations (before and after mid-season, respectively), while the gray area corresponds with the values during the whole treatment season. We considered 15 July as the date for the mid-season split, as it divides in two the treatment season (1 April to 31 October)

We compared the deviations from the mean temperature and mean 21-day cumulative rainfall at the times of treatment for each year, using averages calculated over the treatment period from 1 April to 31 October (Fig. 4). For reference, the average temperatures during the treatment periods from 2020 to 2022 at the botanical garden were 19.8 °C, 19.6 °C, and 21.1 °C, respectively, while the average 21-day cumulative rainfall levels were 54.6 mm, 38.2 mm, and 26.7 mm, respectively. We found significant differences between the distributions of temperature and rainfall deviations at the times of treatment compared with the background distribution in all cases, except for temperature in the $C_{0.50}$ scenario (Table 2). In spring and early summer, optimal treatment deployments occurred at colder-than-average temperatures more frequently than expected based on the background distribution of temperature deviations. Conversely, in late summer and autumn, optimal treatment deployments occurred at warmer-than-average temperatures at a frequency consistent with the background distribution, suggesting that temperature does not play a role in determining optimal treatment times in late summer and autumn. Overall, our results indicated that optimal treatments were more likely to be selected during colder-than-average conditions rather than during warmer-than-average temperatures. In addition, optimal treatments were significantly skewed toward periods of low cumulative rainfall, as these periods were distinctly overrepresented compared with the background deviations. In other words, 21-day cumulative rainfall at treatment times was lower than what would be expected if treatment times were randomly selected throughout the treatment season. Similar results were observed for the $C_{0.75}$ and $C_{max}$ scenarios, which are detailed in Additional File 1.

### Spatial optimization

**Fig. 5:**
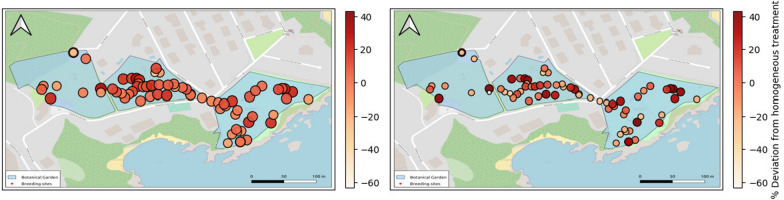
Spatial distribution of treatments for $C_{0.50}$ (left) and $C_{0.25}$ (right). Dot color represents the deviation from an homogeneous treatment for each *C* value, while dot size is proportional to the number of treatments in each breeding site, which is directly related to the value of *C* but also shows spatial variability as also observed in the deviations from homogeneous treatment

Figure 5 illustrates the spatial distribution of treatments from our simulations. Results for the $C_{0.75}$ and $C_{max}$ scenarios are detailed in Additional File 1. Except for the $C_{max}$ scenario, where all breeding sites are treated at every intervention by definition, optimized treatments were not uniformly distributed across breeding sites.

As the amount of larvicide *C* is reduced (e.g., from $C_{0.50}$ to $C_{0.25}$ in Fig. 5), the number of treatments per breeding site is reduced (compare sizes in in Fig.  5 left and right), and the spatial distribution of treatments becomes more heterogeneous, but no clear pattern emerges *a priori* (compare colors in in Fig.  5 left and right). To understand the causes behind the spatial heterogeneity in the optimized treatments, we conducted a logistic regression analysis (see Methods). The best-fitting minimal model contained three log-transformed variables: maximum volume and surface area of breeding sites, and larvicide dose. The logarithm of both maximum volume and surface area of breeding sites showed a positive effect on the probability of treatment, indicating that larger breeding sites are more likely to be treated. Conversely, the logarithm of the larvicide dose exhibited a negative impact on treatment probability, suggesting that higher doses are associated with a lower likelihood of treatment (Table 3). The fact that the log-odds of the outcome variable presents a behavior closer to linear with respect to the logarithmic transformation of the covariates means that the relation of these covariates with these log-odds is better explained by relative change rather than absolute change. In other words, to increase the log-odds by a certain amount, the covariates need to undergo some percentage change. The model’s accuracy, defined as the proportion of correct predictions out of the total observations, was 68.52%. The confusion matrix detailing the fit of the model is provided in Additional File 1.

Despite the exclusion of space-related variables during the step-wise backward deletion process owing to their lack of significance, we assessed potential spatial autocorrelation in the optimized treatment outcomes by computing Moran’s Index for the three values of *C*. The results indicated no significant spatial autocorrelation, with *p*-values of 0.27, 0.10, and 0.16 for $C_{0.25}$, $C_{0.50}$, and $C_{0.75}$, respectively.

**Fig. 6:**
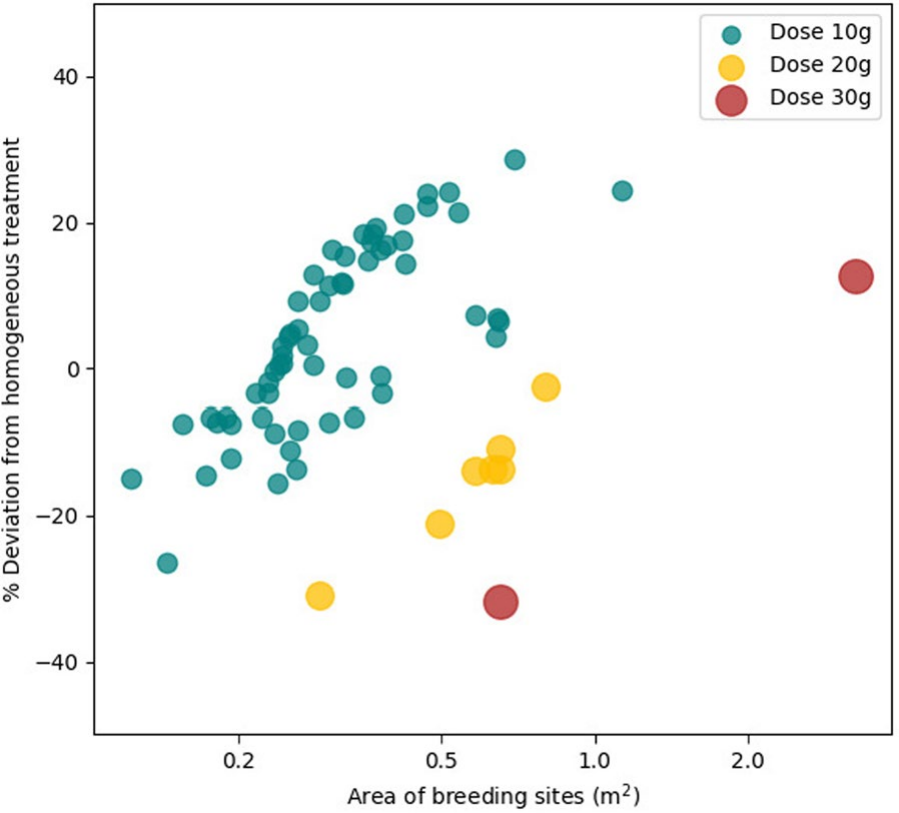
Relative treatment frequency of breeding sites with respect to an homogeneous control strategy, represented against their area in a Napierian logarithmic scale, averaging simulations for $C_{0.25}$, $C_{0.50}$, and $C_{0.75}$. Positive values represent increased number of treatments in percentage respect to the homogeneous control strategy, and vice versa for negative values

Figure 6 provides another way to understand the results of Table 3. There, we observe a linear positive influence of the logarithm of the area of the breeding site on the deviation from homogeneous treatment (computed as the number of treatments performed in a breeding site in comparison with what would be expected if they were all treated equally). This tendency holds within treatment dose groups, but breaks when considering breeding sites from different dose groups. Breeding sites with the same area are less frequently treated if they are treated at a higher dosage.

### Optimized *versus* routine vector control strategies

We evaluated the cost-effectiveness of optimized compared with routine vector control practices at the botanical garden. Routine practices involved treatment applications at regular intervals throughout the season, similar to the current control strategies at the garden. To compare both strategies, we recreated a typical routine strategy which consisted of four treatments, spaced 7 weeks apart. The interval was chosen on the basis of the efficacy of Vectomax^®^ FG found in previous studies in the botanical garden [16]. Given that the mosquito season spans from 1 April to 31 October, the first of the four routine treatments could be scheduled any day between 1 April and 18 April to ensure all four treatments are completed by the end of the season.

To fully characterize the routine strategy, it is necessary to specify both treatment timing and the breeding site selection. For the routine strategy, the breeding sites with the highest larval activity were prioritized for each intervention. The larvicide dosage used at each site matched that of the optimized strategies, 10 g, 20 g, or 30 g depending on the site’s dose group. In addition, the larvicide was evenly distributed across the four treatments, with one-quarter of the total product used per treatment.

To compare the efficacy of both strategies, optimized (*opt*) and routine (*rout*), we computed the percentage of reduction in the mosquito population achieved, $\Delta J^{\text {opt}}$ and $\Delta J^{\text {rout}}$, for different amounts of larvicide, *C*, averaging across the years 2020, 2021, and 2022. Formally, we defined this quantity as $\Delta J^{\text {opt}}:=100\cdot \frac{J^{\text {opt}}-J^0}{J^0}$, where *J* is given by $J:=\int _0^{t_f}\left( \sum _{i=1}^{N}A_i(t) \right) dt$, and $J^0$ refers to the value of *J* when no control is applied.

We conducted 25 simulations for each year and *C* value (300 in total). In each simulation, the date for the first treatment was randomly selected from 1 April to 18 April. Table 4 shows the results for the average reduction across all years and simulations ($\Delta J^{\text {rout,a}}$), and the greatest reduction achieved in any simulation ($\Delta J^{\text {rout,b}}$) for each *C* value.

**Fig. 7:**
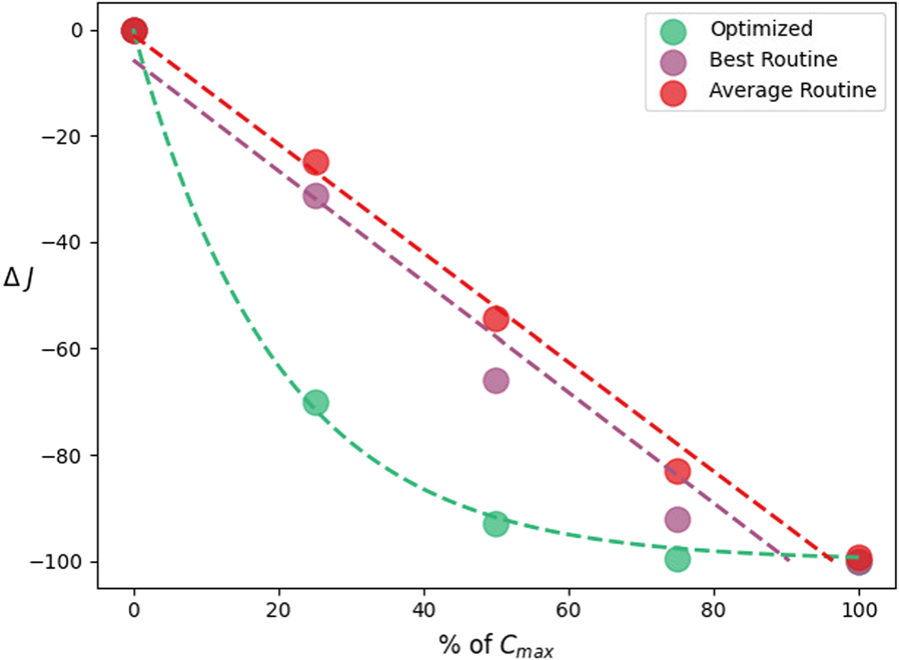
Average mosquito percentage reduction in abundance against the amount of larvicide deployed (as a percentage of $C_{max}$) for optimized and routine strategies in the botanical garden and their respective best fits

**Fig. 8:**
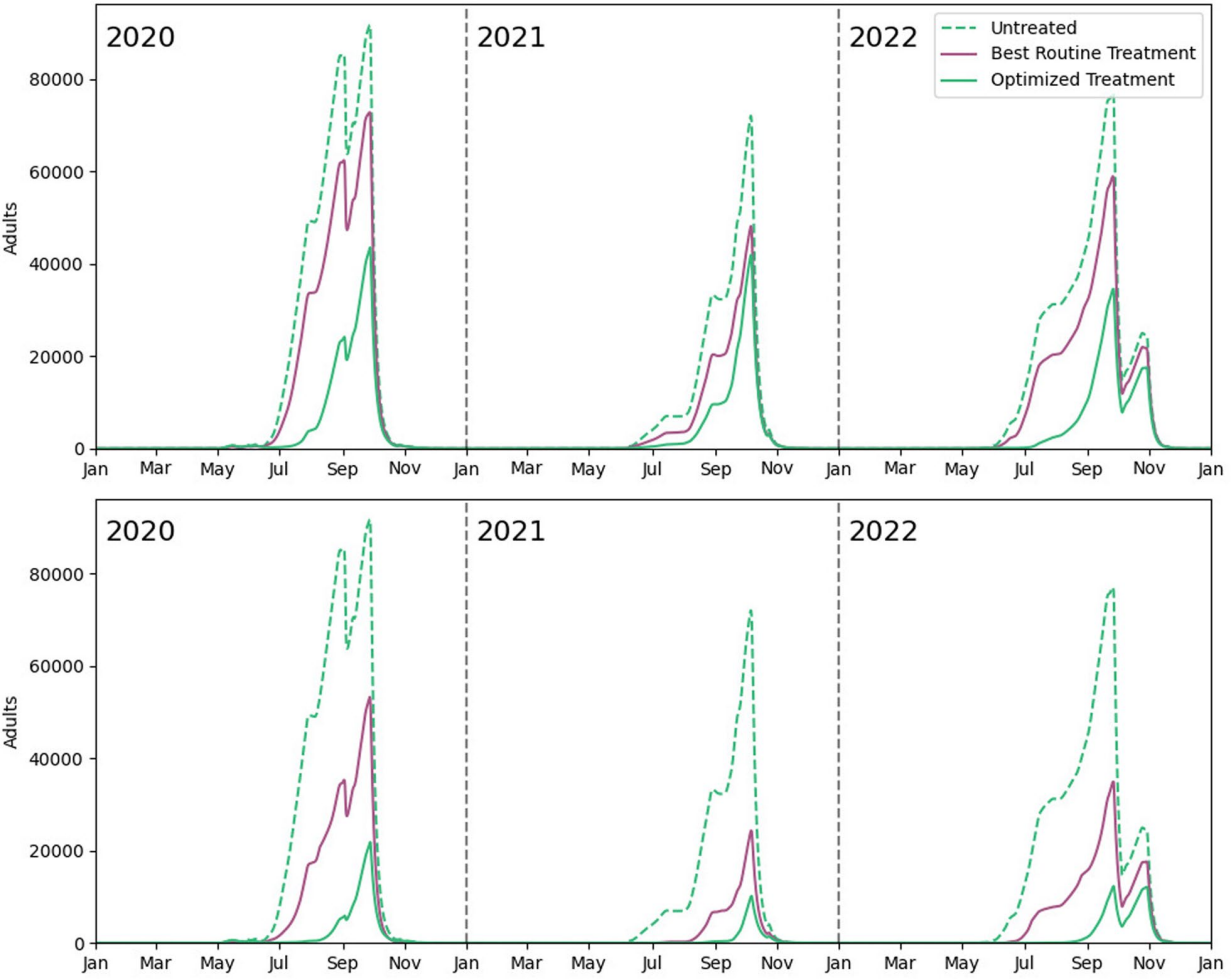
Adult mosquito population dynamics in the botanical garden for routine and optimized vector control strategies for $C_{0.25}$ (top) and $C_{0.50}$ (bottom)

For high amounts of larvicide ($C = C_{max}$ and $C=C_{0.75}$), both the optimized and routine strategies performed similarly. However, notable differences emerged for lower amounts of larvicide ($C = C_{0.50}$ and more markedly for $C = C_{0.25}$). The optimized strategy significantly outperformed the routine strategy in reducing adult mosquito populations when less larvicide was used, as demonstrated in Fig. 7 and Fig. 8.

To quantify this, we fit the $\Delta J$ values obtained across all *C* values for the strategies explained. For the optimized strategy ($\Delta J^{\text {opt}}$), the percentage decrease in adult mosquito population was exponential with *C*. In contrast, for the average routine strategy ($\Delta J^{\text {rout,a}}$), the mosquito reduction was linear with *C*. These claims are backed by both the $R^2$ of each fit and by the differences in the Akaike information criterion (AIC) between the best fit and its alternative.^1^ When considering only the best simulation in the routine strategy scenario for each *C* value ($\Delta J^{\text {rout,b}}$), the linear model still fits best, but the difference in the AIC is not large enough to dismiss the exponential model. $R^2$ values and AIC differences are shown in Table 5.

## Discussion

The invasive nature of the mosquito *Ae. albopictus* and its adaptation to densely populated urban areas in Europe, where pathogens can be easily imported from endemic regions, heighten epidemiological risks. In this context, even marginal improvements in mosquito control become highly significant. Using a mathematical model, we represented the population dynamics of *Ae. albopictus* in a botanical garden. Our model accounted for fluctuations in local mosquito populations driven by key environmental variables (e.g., temperature and rainfall), the specific spatial configuration and characteristics of water pools, and human vector control efforts. This approach allowed us to assess the impact of larvicidal control on mosquito populations throughout the year and to develop climate-responsive practices for mosquito control.

We found that larvicide treatments are more effective when applied during periods of lower cumulative rainfall compared with the seasonal average (Fig. 4). This increased effectiveness can be attributed to two main factors: first, smaller water volumes support fewer larvae, requiring less larvicide for control. Second, reduced water volumes lead to higher larvicide concentrations per unit of water. This effect is especially significant in resource-scarce vector control scenarios. Moreover, our data suggest that optimal treatment timing often occurs early in the season (Fig. 3), suggesting that treatments are more effective when larval populations are still small. Adverse climatic conditions during winter naturally keep mosquito populations low. However, improved conditions at the beginning of the season can lead to rapid population growth. Therefore, early intervention is crucial to prevent population surges as cumulative rainfall increases and breeding sites become more abundant. Later in the season, extreme heat and lack of rainfall can lead to natural population crashes, followed by spikes in mosquito numbers after late-summer rainfall events. Our simulations indicate that targeting these low population periods with treatments can maximize their effectiveness. In other words, to achieve optimal results, treatments should be preventive rather than reactive.

Optimal treatments, according to our results, were more likely to be performed during periods of adverse climatic conditions for larvae development, that is, timings with colder-than-average temperatures and lower-than-average cumulative rainfall. Nonetheless, low rainfall was found to be more influential than cold temperatures, as many optimal treatments occurred also at warmer-than-average temperatures. The onset of water accumulation and potential breeding site proliferation, occurring early in the mosquito season, seemed to be the primary triggering factor for vector control. By linking climatic fluctuations to optimal treatment timing, our findings enhance the potential flexibility of treatment schedules while keeping or improving efficacy. Of note, forecasts of temperature and rainfall 7–15 days in advance could support more proactive and effective vector control planning.

Since no spatial autocorrelation was detected in treatment probability, we concluded that treatments should be based solely on breeding site characteristics, such as size and shape, rather than their spatial location. This conclusion is supported by the fact that variables related to the spatial distribution of breeding sites were of low significance and were excluded during model selection in our regression analysis. Two factors could obscure potential spatial effects: homogeneity in breeding site distribution and a spatial scale that may be too small relative to adult mosquito mobility. The first factor can be ruled out in our case. This is so, because in our model, the dispersion rate of adult mosquitoes is based solely on pairwise distances between breeding sites. The distribution of distances among breeding sites is not homogeneous, as the botanical garden is divided in three interconnected but relatively distanced sections. Regarding the second, estimates of average distances traveled by *Ae. albopictus* mosquitoes in urban areas are around 160 m [30, 32], which is approximately the size of each one of the three sections in which the garden is divided. This indicates significant dispersal between most breeding sites, leading to spatial homogenization of adult populations and subsequent water-pool colonization. Therefore, while the specific spatial distribution of breeding sites may not be critical in our current setting, it could become more relevant in larger or differently structured environments.

Our findings suggest that prioritizing larger breeding sites for treatment can be more efficient, as these sites, which accumulate more water, tend to produce a higher number of mosquito larvae. Consequently, targeting such sites has the potential to have a greater impact on reducing the mosquito population. However, in situations where budgetary or operational constraints exist, there is a trade-off between breeding site size and treatment cost, as larger sites often require higher dosages, increasing treatment expenses. This highlights the importance of a proper matching between larval production and treatment dose. Treating large sites with excessive dosages may result in diminishing returns, which makes it more effective to distribute treatment across multiple smaller sites that collectively contribute more significantly to mosquito population control.

The results from our study demonstrate that adapting a vector control strategy to the weather conditions and characteristics of a study site is significantly more cost-effective than adopting a routine strategy, especially at lower larvicide doses. As shown in Table 4, for lower larvicide quantities (e.g., $C_{0.25}$ and $C_{0.50}$), the optimized approach consistently outperformed the routine method in reducing the mosquito population. This difference was particularly notable at $C_{0.25}$, where the optimized strategy achieved a 70.19% reduction compared with reductions of 25–30% in the routine cases. Although for higher amounts of larvicide ($C_{0.75}$ and $C_{max}$) both strategies converged to near-complete elimination of mosquitoes, the fact that the optimized approach reduces the adult mosquito population exponentially as resources increase, as opposed to the linear reduction observed with the routine strategy (more prominently in the averaged case), underscores the fact that tailored approaches should be preferred. We remark that we only considered for this analysis the cost of the product, neglecting other expenses such as salaries or operational costs, which, in our setting, are not expected to differ significantly between the two strategies.

Finally, it is important to approach our results with caution, as vector control in real-world settings involves numerous factors beyond climate variation, dispersal, and breeding site characteristics that are not accounted for in our model. Some limiting factors of this study are technical specifications of larvicides and local conditions affecting their efficacy [43–46], the complex ecology of urban mosquitoes [47] or the intricate relationship between rainfall patterns, water pool availability, and treatment effectiveness [16, 27]. Namely, we did not include flushing effects induced by rainfall or the formation of secondary breeding sites that can be colonized by *Ae. albopictus* females when the highly productive ones are not available [48]. Finally, another extension of our model could consider overcompensation effects [49], as it has been documented that in some cases the stress induced by an external mortality force can trigger an overall population increase.

## Conclusions

We leverage mathematical modeling and statistical analysis to propose an innovative methodology for assessing and optimizing resource allocation to develop best practices for mosquito control. Through *in silico* simulations, we demonstrate that optimizing larvicide application by incorporating weather variables and the intrinsic characteristics of breeding sites can significantly enhance the effectiveness of periodical treatments. Simple adjustments to treatment practices, especially when only a limited number of breeding sites are treated owing to budgetary or operational constraints, can lead to substantial reductions in target mosquito populations. While our results may not directly apply to scenarios such as large cities, where mosquito dispersal includes additional factors such as vehicle transport, they provide a solid foundation for refining treatment policies in diverse settings, offering general guidelines applicable to breeding site collections where rainfall influences water dynamics and where mosquitoes primarily disperse on their own. Moreover, the mathematical framework presented can be a valuable tool for future research in various contexts, provided it is adapted appropriately.

## Supplementary Information

Below is the link to the electronic supplementary material.

Supplementary file 1: Fig. S1. Treatment densities and average larva dynamics for C0.75 and Cmax Fig. S2. Deviation from mean temperature and mean 21-day cumulative rainfall at treatment for Cmax and C0.75. Fig. S3. Spatial distribution of treatments for Cmax and C0.75. Table S1. Confusion matrix of best-fitting minimal GLMM. Fig. S4 Adult mosquito population dynamics in the botanical garden for routine and optimized vector control strategies, for C0.75 and Cmax. Additional file 2: Text S1. Details on larvicide’s residual activity bioassays Table S2. Number of dead larvae found in the containers after the application of different doses. Fig. S5. Graphical representation of the larval percent mortality obtained from the bioassay with the different treatment dosages. Additional file 3: Text S2. Comments on vector-borne disease transmission minimization Fig. S6. Mean temperature, 21-day cumulative precipitation and daily visitors at the botanical garden Marimurtra on 2021 and 2022. Fig. S7. Treatment densities and average larva dynamics for cases C0.25 and C0.50, when minimizing adult mosquitoes and nuisance for visitors/vector-borne disease transmission

## Data Availability

The data and code used in this article can be found in the public repository: https://github.com/jesusbellver/Optimizing-Larvicide-Treatment-Strategies-in-a-Botanical-Garden.git

## Footnotes

1 An AIC difference greater than 10 between the best fitting model and the alternative indicates no empirical support for the alternative model.
