# Supplementary material for: Climate-responsive vector control strategies for Aedes albopictus
Source: Parasit Vectors. 2025 May 11;18:168. doi: 10.1186/s13071-025-06791-2 (PMC12067949; doi:10.1186/s13071-025-06791-2)
Supplement: Supplementary file 1 — Fig. S1. Treatment densities and average larva dynamics for C0.75 and Cmax Fig. S2. Deviation from mean temperature and mean 21-day cumulative rainfall at treatment for Cmax and C0.75. Fig. S3. Spatial distribution of treatments for Cmax and C0.75. Table S1. Confusion matrix of best-fitting minimal GLMM. Fig. S4 Adult mosquito population dynamics in the botanical garden for routine and optimized vector control strategies, for C0.75 and Cmax. Additional file 2: Text S1. Details on larvicide’s residual activity bioassays Table S2. Number of dead larvae found in the containers after the application of different doses. Fig. S5. Graphical representation of the larval percent mortality obtained from the bioassay with the different treatment dosages. Additional file 3: Text S2. Comments on vector-borne disease transmission minimization Fig. S6. Mean temperature, 21-day cumulative precipitation and daily visitors at the botanical garden Marimurtra on 2021 and 2022. Fig. S7. Treatment densities and average larva dynamics for cases C0.25 and C0.50, when minimizing adult mosquitoes and nuisance for visitors/vector-borne disease transmission [file 13071_2025_6791_MOESM1_ESM.pdf]

Additional File 1: Supporting graphs and tables

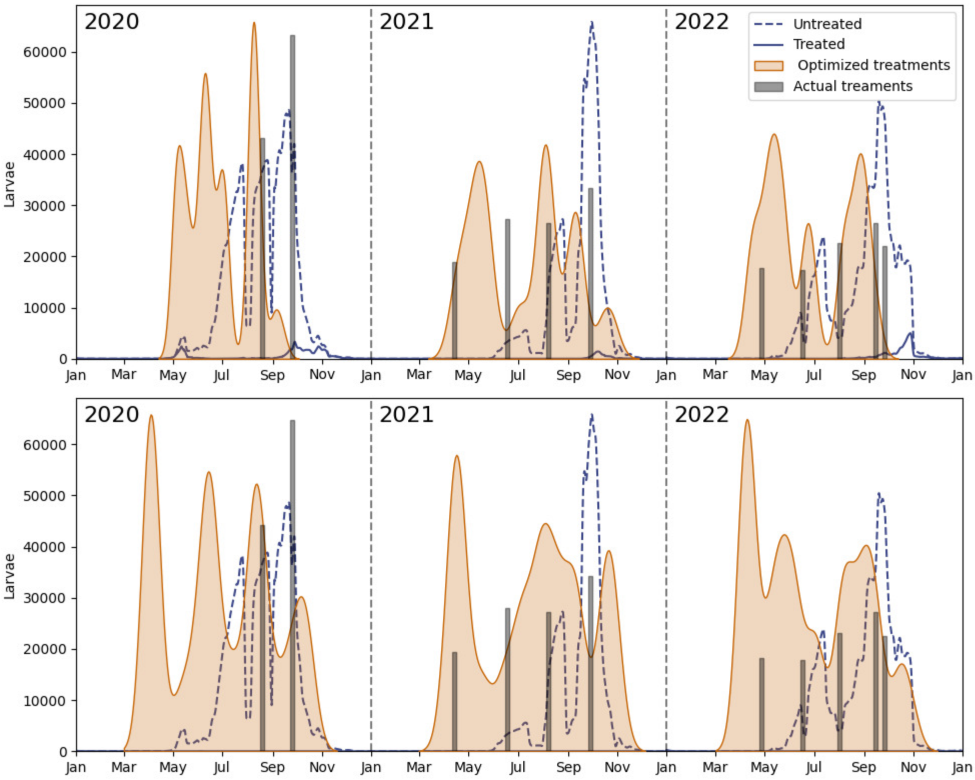

Fig. S1: Treatment densities and average larva dynamics for $C_{0.75}$ (top) and C_{max} (bottom). The actual distribution of treatments performed in the botanical garden was included for reference.

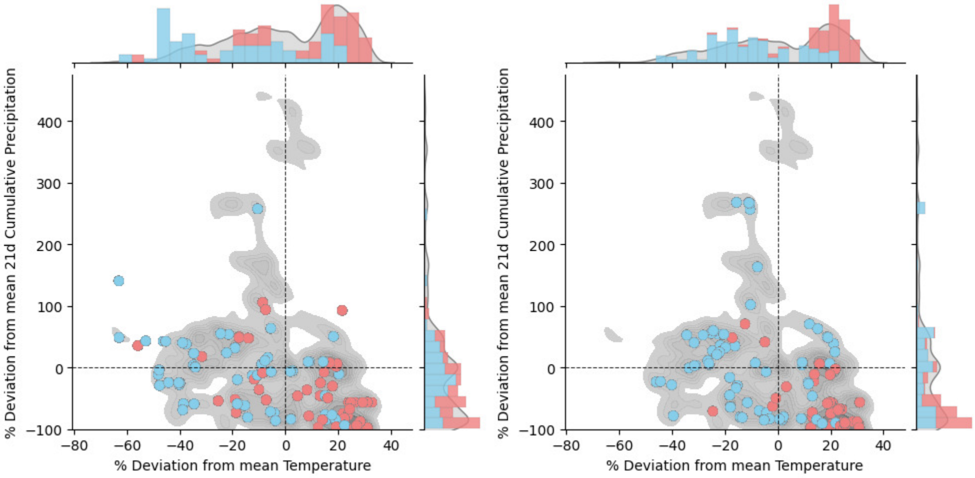

Fig. S2: Deviation from mean temperature and mean 21-day cumulative rainfall at treatment for C_{max} (left) and $C_{0.75}$ (right). Blue and red dots correspond with treatment times resulting from simulations (before and after mid-season respectively), while the gray area corresponds with the values during the whole treatment season. We considered the 15th of July as the date for the mid-season split, as it divides in two the treatment season (1st April - 31st October).

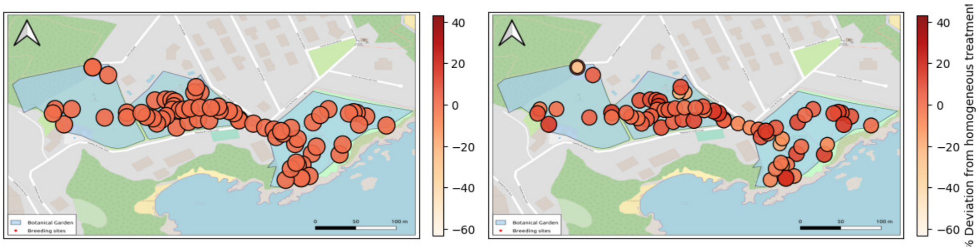

Fig. S3: Spatial distribution of treatments for C_{max} (left) and $C_{0.75}$ (right). Dot color represents the deviation from an homogeneous treatment, while dot size is proportional to the number of times a breeding site is treated across the simulations analysed.

Actual \ Predicted	0	1
0	**8077**	4250
1	3683	**9190**

Table S1: Confusion matrix of best-fitting minimal GLMM.

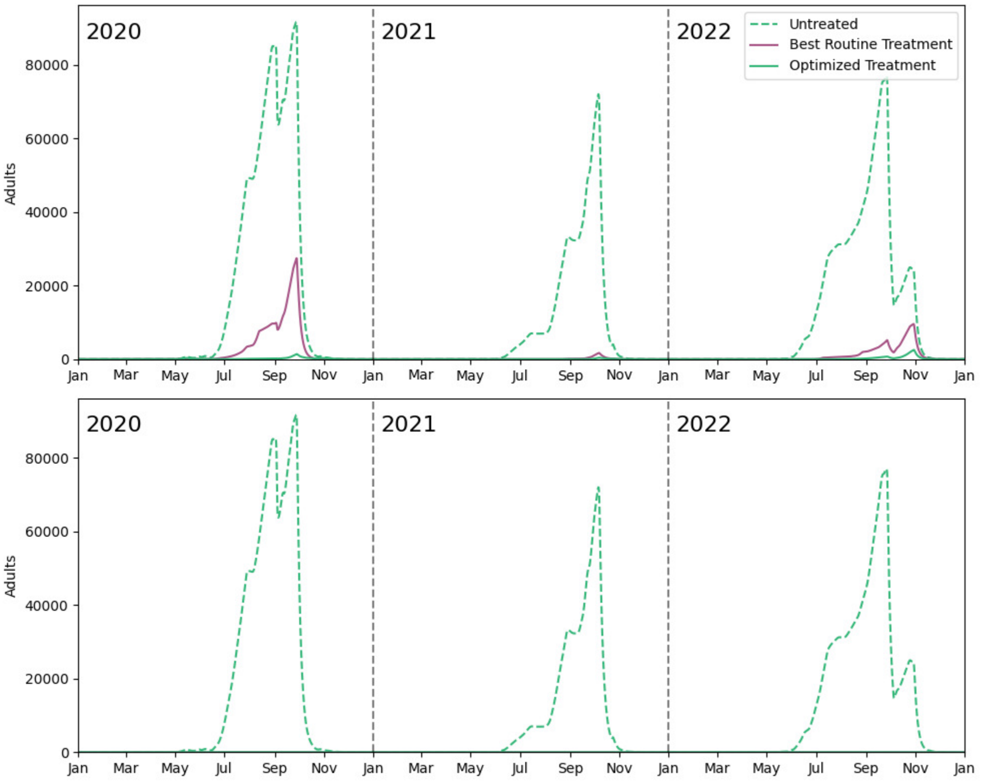

Fig. S4: Adult mosquito population dynamics in the Botanical Garden for routine and optimized vector control strategies, for $C_{0.75}$ (top) and C_{max} (bottom).

Additional File 2: Larvicide's residual activity bioassays

We conducted a bioassay in laboratory conditions, at temperatures between 20 °C and 25 °C, to gather data on the residual activity of Vectomax® FG in order to fit the following system's parameters: the larvicide's toxicity, τ, its intrinsic decay rate, κ_c, and the active removal rate by the larvae, κ_L.

To do so, we arranged nine 0.5L liquid sample collection containers into three groups: Treated, Over-treated and Control. Each container held 0.5L of water and 50mg of organic matter (smashed oak leaves) as a source of food. We applied 100mg of Vectomax per container in the Treated group, recommended dosage by the manufacturer, 335mg in the Over-treated group, equivalent to the maximum dosage used by the company in charge for the treatments (see main text), and 0g for the control group. Starting at week 1 (first week of August 2021) and for 11 weeks, at intervals of one week approximately, we introduced five *Ae. albopictus*' larvae inside each one of the containers and we recorded their mortality several times (10 measures) in the following 24h [1]. After 24h all larvae were removed, whether dead or alive, until the following week in which the survival analysis was repeated. We added one additional set of 3 containers in the Treated group (i.e. 100mg of Vectomax) in which we only introduced 5 larvae of *Ae. albopictus* once, 56 days (8 weeks) after the application of the larvicide. Then, we evaluated survival over the subsequent 24 hours. The goal of these extra containers was to be able to differentiate with greater precision the difference between the intrinsic decay rate of the product and the one associated to the ingestion by the larvae. Results can be found in Table S2 and Figure S5.

To fit the parameters of interest we considered an ODE system which can be derived from the main system studied in this work. The derivation is done by not taking adults into account and by assuming the following simplifying assumptions: development into adults is negligible at the time scales of the bioassay (no larva is left in the container more than 24h), larval death only occurs due to the larvicide's effect and intraspecific competition for resources is negligible in the containers. These assumptions are reasonable in the context of the bioassay. Taking this into account, we write the following ODE system:

$$\begin{aligned} \frac{dL}{dt} &= -\tau \frac{cL}{V_0}, \\ \frac{dc}{dt} &= -\kappa_c c - \kappa_L \frac{cL}{V_0}. \end{aligned} \tag{1}$$

Larvicide dose (mg)	Days since application	1h	2h	3h	4h	5h	6h	7h	8h	9h	24h
0	0	0	0	0	0	0	0	0	0	0	0
	8	0	0	0	0	0	0	0	0	0	0
	14	0	0	0	0	0	0	0	0	0	0
	22	0	0	0	0	1	1	1	1	1	1
	28	0	0	0	0	0	0	0	2	2	4
	36	0	0	0	0	0	0	0	0	0	0
	42	0	0	0	0	0	0	0	0	0	0
	50	0	0	0	0	0	0	0	0	0	0
	56	0	0	0	0	0	0	0	0	0	0
	63	0	0	0	0	0	0	0	0	0	0
	70	0	0	0	0	0	0	0	0	0	0
	77	0	0	0	0	0	0	0	0	0	0
100	0	12	12	14	14	14	14	14	14	14	15
	8	6	14	14	14	14	14	14	14	14	15
	14	4	8	12	14	14	15	15	15	15	15
	22	1	4	5	11	13	13	13	13	13	15
	28	0	4	11	12	14	14	14	15	15	15
	36	0	1	3	5	9	11	11	13	13	15
	42	0	0	0	3	4	6	8	8	8	13
	50	0	0	0	0	0	0	0	1	1	3
	56	0	0	0	0	0	0	0	0	0	0
	56*	0	5	7	10	10	11	12	12	12	15
	63	0	0	0	0	0	0	0	1	1	5
	70	0	0	0	0	0	0	0	0	0	3
	77	0	0	0	0	0	1	1	1	1	1
335	0	13	15	15	15	15	15	15	15	15	15
	8	12	15	15	15	15	15	15	15	15	15
	14	6	14	15	15	15	15	15	15	15	15
	22	1	8	10	12	13	14	14	14	14	15
	28	3	6	13	15	15	15	15	15	15	15
	36	1	1	4	8	10	14	14	14	14	15
	42	0	3	4	9	9	9	11	12	13	15
	50	0	0	0	0	0	0	0	0	1	12
	56	0	1	1	1	1	1	2	2	3	12
	63	0	0	0	0	0	0	0	0	0	7
	70	0	0	0	1	1	1	1	1	1	8
	77	0	0	0	0	0	0	0	1	1	6

Table S2: Number of dead larvae found in the containers after the application of different doses. The row 56* corresponds to the additional containers where no larvae had been previously placed.

We use this system to reproduce dynamics of the larvae and the larvicide in the containers for the different scenarios of the bioassay. In this case $V_0 = 0.5$l and does not vary with time. We fix an initial treatment amount, for instance, $c(0) = 100$mg, and we consider an introduction of 15 larva, $L(0) = 15$, five for each of the three containers. Any surviving larva is removed after 1 day ($L(1) = 0$). This discontinuities are repeatedly applied at the subsequent dates ($L(8) = 15$, $L(9) = 0$ and so on). We simulated the same dynamics for $c(0) = 335$mg and for the case $c(0) = 100$mg, but with only one introduction of larvae at day (row 56* in Table S2). We adjusted the values of τ, κ_c, and κ_L so that system (1) reproduces as well as possible the data in Table S2. The control case was not simulated since it was only carried to ensure the absence of any anomalous source of mortality.

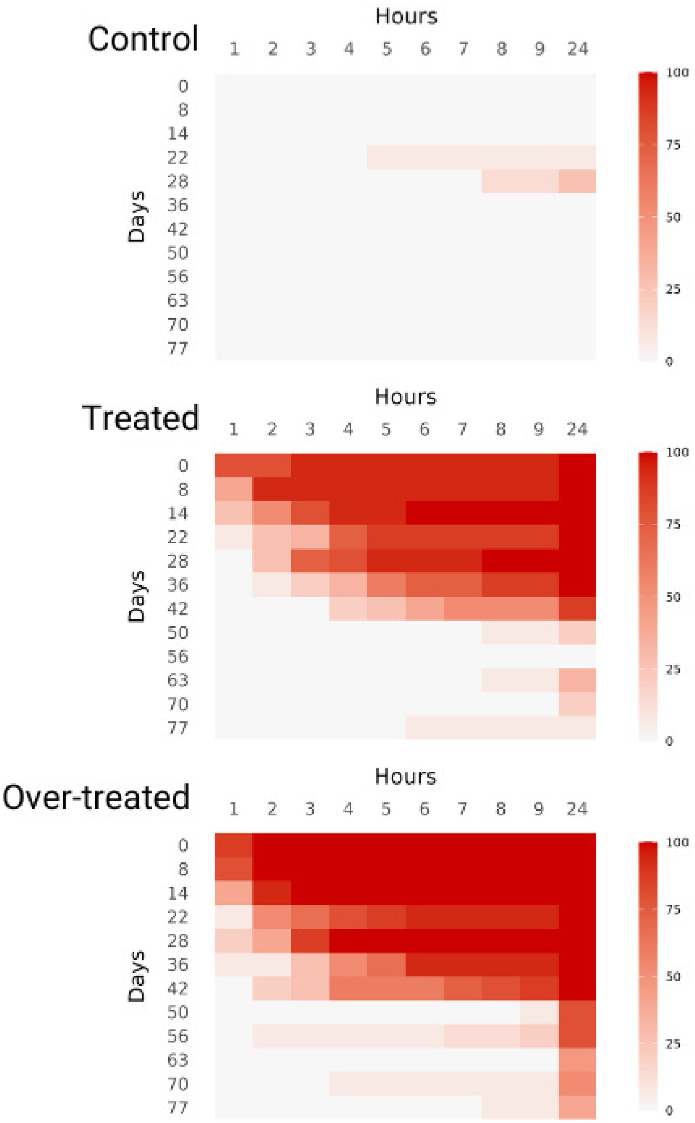

Fig. S5: Graphical representation of the larval percent mortality obtained from the bioassay with the different treatment dosages. The hours after the larvae placement in the containers (x-axis) and the days after the treatment application (y-axis) are shown.

References

[1] WHO: Guidelines for Laboratory and Field Testing of Mosquito Larvicides. World Health Organization, Geneva (2005)

Additional File 3: Vector-borne disease transmission minimization

In this study, we investigated optimal larvicide treatment strategies for controlling mosquito populations in a botanical garden. By "optimal", we specifically referred to strategies that minimize the adult mosquito population throughout the year. However, it is important to note that this criterion is not the only valid measure of optimality. An alternative definition focuses on minimizing the nuisance experienced by visitors, which involves reducing the number of mosquito-human interactions. This approach is particularly relevant in minimizing the potential transmission of vector-borne diseases.

To model this alternative criterion, we can weight the adult mosquito population by the number of visitors present in the garden at any given time. In other contexts, this methodology could be adapted to public spaces or residential areas by considering the number of daily users or residents throughout the year. Let $v(t)$ represent the number of visitors at time t. Under this framework, the optimization problem can be reformulated as:

$$\inf_{\substack{\{t_k\}_k \in \mathcal{T} \\ \{\xi_k^i\}_{i,k} \in \Gamma}} \int_0^{t_f} v(t) \sum_{i=1}^{N} A_i(t) dt. \tag{1}$$

The mathematical model governing mosquito population dynamics is the the same as the one detailed in the primary manuscript (see System (3) therein).

To address this problem numerically, we employed the same methodology outlined in the main manuscript. We performed simulations for $C = C_{0.25}$ and $C = C_{0.50}$, since, in these scenarios, resource optimization becomes more relevant. Furthermore, we restricted simulations to the years 2021-2022, as these were the only years for which daily visitor data for the botanical garden was available. Figure S6 presents this data alongside the 21-day cumulative precipitation and mean temperature for these years, providing context for the environmental conditions during the study period.

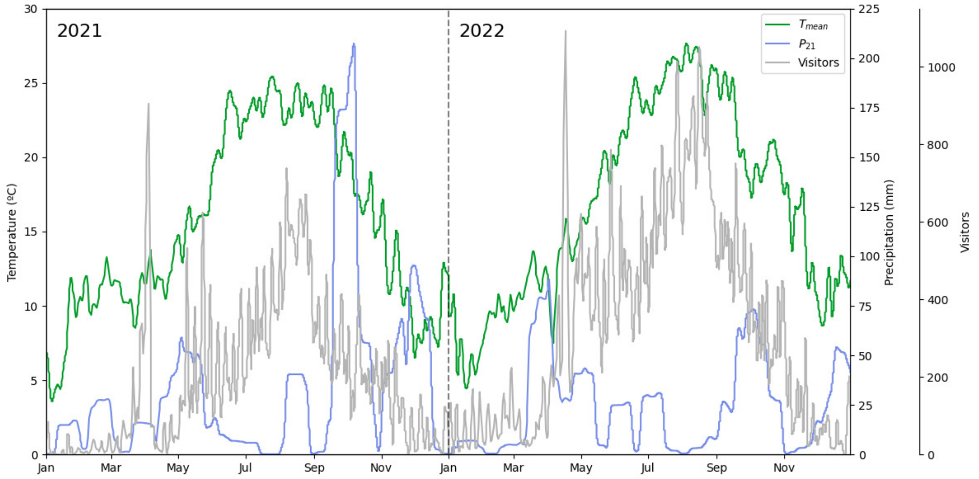

Fig. S6: Mean temperature, 21-day cumulative precipitation and daily visitors at the botanical garden Marimurtra on 2021 and 2022.

The results obtained do not differ substantially from those obtained by minimizing the overall mosquito population (See Figure S7). As in the other case, treatments are primarily concentrated in late spring to early summer in the $C_{0.25}$ case, while both cases show the highest treatment intensities occurring during periods of potential larval population surges. This suggests that directly targeting the mosquito population may be sufficient to achieve significant reductions in vector-borne disease transmission, without requiring more complex control strategies.

One possible explanation for this similarities lies in the temporal correlation between visitor numbers and mean temperature, as shown in Figure S6. With the exception of the Easter holiday period, touristic activity tends to be higher during the austral summer, when temperatures are warmer in the northern hemisphere. Given that mosquito populations are also temperature-dependent, the periods of higher visitor numbers coincide with the periods of bigger mosquito populations. Although the mosquito population peak is slightly skewed toward autumn due to several concurrent factors (mainly rainfall), this overlap means that, from a mathematical perspective, minimizing the total mosquito population and minimizing visitor-mosquito contact yield similar outcomes. The Easter holiday, despite its surge in visitor numbers, occurs too early in the mosquito treatment season to have a meaningful impact on control efforts. As a result, it does not significantly influence the results.

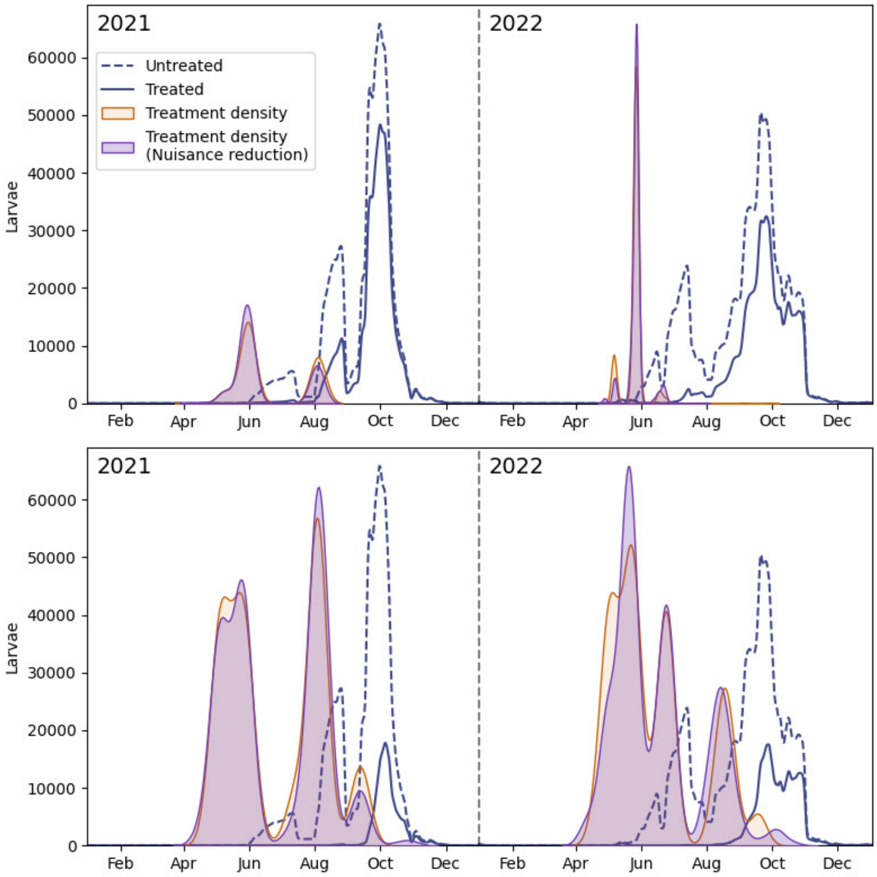

Fig. S7: Treatment densities and average larva dynamics for cases $C_{0.25}$ (top) and $C_{0.50}$ (bottom), when minimising adult mosquitoes (orange) and nuisance for visitors - v.b.d. transmission (blue).
